# Arrhythmogenesis in Timothy Syndrome is associated with defects in Ca^2+^-dependent inactivation

**DOI:** 10.1038/ncomms10370

**Published:** 2016-01-29

**Authors:** Ivy E. Dick, Rosy Joshi-Mukherjee, Wanjun Yang, David T. Yue

**Affiliations:** 1 Calcium Signals Laboratory, Departments of Biomedical Engineering and Neuroscience, The Johns Hopkins University School of Medicine, Ross Building, Room 713, 720 Rutland Avenue, Baltimore, Maryland 21205, USA

## Abstract

Timothy Syndrome (TS) is a multisystem disorder, prominently featuring cardiac action potential prolongation with paroxysms of life-threatening arrhythmias. The underlying defect is a single *de novo* missense mutation in Ca_V_1.2 channels, either G406R or G402S. Notably, these mutations are often viewed as equivalent, as they produce comparable defects in voltage-dependent inactivation and cause similar manifestations in patients. Yet, their effects on calcium-dependent inactivation (CDI) have remained uncertain. Here, we find a significant defect in CDI in TS channels, and uncover a remarkable divergence in the underlying mechanism for G406R versus G402S variants. Moreover, expression of these TS channels in cultured adult guinea pig myocytes, combined with a quantitative ventricular myocyte model, reveals a threshold behaviour in the induction of arrhythmias due to TS channel expression, suggesting an important therapeutic principle: a small shift in the complement of mutant versus wild-type channels may confer significant clinical improvement.

## Abstract (web summary)

Timothy Syndrome (TS) is a multisystem disorder caused by two mutations leading to dysfunction of the Ca_V_1.2 channel. Here, Dick *et al*. uncover a major and mechanistically divergent effect of both mutations on Ca^2+^/calmodulin-dependent inactivation of Ca_V_1.2 channels, suggesting genetic variant-tailored therapy for TS treatment.

## Graphical abstract

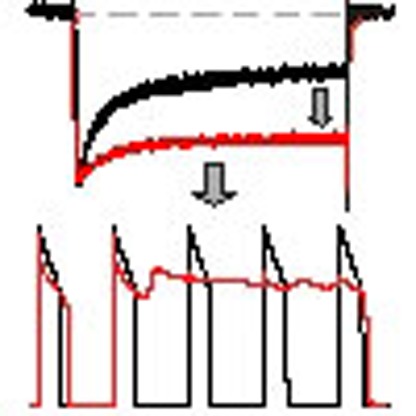

Timothy Syndrome (TS) is a multisystem channelopathy in which patients suffer from long QT syndrome, immune deficiencies and autism^1^,^2^. TS presents in two major forms differing primarily in their TS channel expression pattern^1^. In TS type 1, a G406R mutation occurs within the mutually exclusive exon 8a (using the nomenclature of Splawski *et al*.^1^,^2^) splice variant of Ca_V_1.2. This variant is expressed in a wide distribution of tissues, but represents only 20% of Ca_V_1.2 channels in the heart^2^,^3^. This explains the multisystem nature of TS type 1, which includes prolonged QT intervals, syndactyly, immune deficits, developmental delays and often autism^2^. However, the lower expression profile of TS type 1 in the heart and brain seems to be more compatible with survival, and thus accounts for the majority of reported cases of TS. In TS type 2, on the other hand, the mutation (either G406R or G402S) occurs within the alternate exon 8, which is expressed in a smaller subset of tissues, but represents the more prominent splice variant in the heart (80% of cardiac Ca_V_1.2)^1^,^4^. TS type 2 patients are rare, but cases have been found for both the G406R and G402S variants. As a result of these differing expression profiles, TS type 2 patients lack syndactyly while displaying more severe cardiac deficits, with profoundly prolonged QT intervals^1^,^5^.

In both variants of TS, the mutations cause a disruption in the function of the Ca_V_1.2 voltage-gated Ca^2+^ channel. These channels are critical conduits of Ca^2+^ entry into the heart, smooth muscle and brain. As such, Ca_V_1.2 channels employ two forms of feedback regulation—voltage-dependent inactivation (VDI) and Ca^2+^/calmodulin-dependent inactivation (CDI). Disruption of these regulatory mechanisms in various Ca^2+^ channels is known to result in severe clinical phenotypes including autism, migraine, night blindness and ataxia^1^,^2^,^6^,^7^,^8^, thus understanding the effects of the TS mutations on both these regulatory mechanisms may provide significant insight into the pathogenesis of TS. Both TS mutations (G406R and G402S) are located within the IS6 region of Ca_V_1.2 channels (Fig. 1a)^1^,^2^ and correspond to a known structural component of VDI^9^,^10^. The most widely reported biophysical consequence of the TS mutations on Ca_V_1.2 is a loss of VDI (refs 1, 2, 11) (Supplementary Fig. 1). However this VDI deficit alone may not represent the full pathology of TS patients^12^,^13^,^14^,^15^. In the heart, CDI plays a particularly important role in setting the action potential duration (APD)^16^,^17^,^18^ and disruption of this vital feedback mechanism is known to result in severe long QT syndrome^19^. Thus, the cardiac pathophysiology seen in TS patients is consistent with a deficit in CDI. However, the biophysical evidence for a CDI defect in TS channels has been equivocal. While one study notes that the G406R TS mutation causes a reduction of CDI in the rabbit Ca_V_1.2 channel^20^, a later study done in the human Ca_V_1.2 channel concludes that the G406R mutation has no effect on the extent of CDI (ref. 11). As CDI may actually play a larger role than VDI in generating cardiac arrhythmias^16^,^17^,^21^, it is important to understand what effect, if any, the TS mutations have on CDI.

In this work, we demonstrate that both TS mutations disrupt CDI, however they do so with distinct mechanisms. Modelling this disruption of CDI predicts profound AP prolongation which is dependent on the fraction of TS channels expressed as well as on the specific underlying mutation (G406R or G402S). This result is verified in adult ventricular myocytes, establishing a threshold behaviour in the induction of arrhythmias due to TS mutations.

## Results

### TS mutation effects on calcium-dependent inactivation

**Figure 1:**
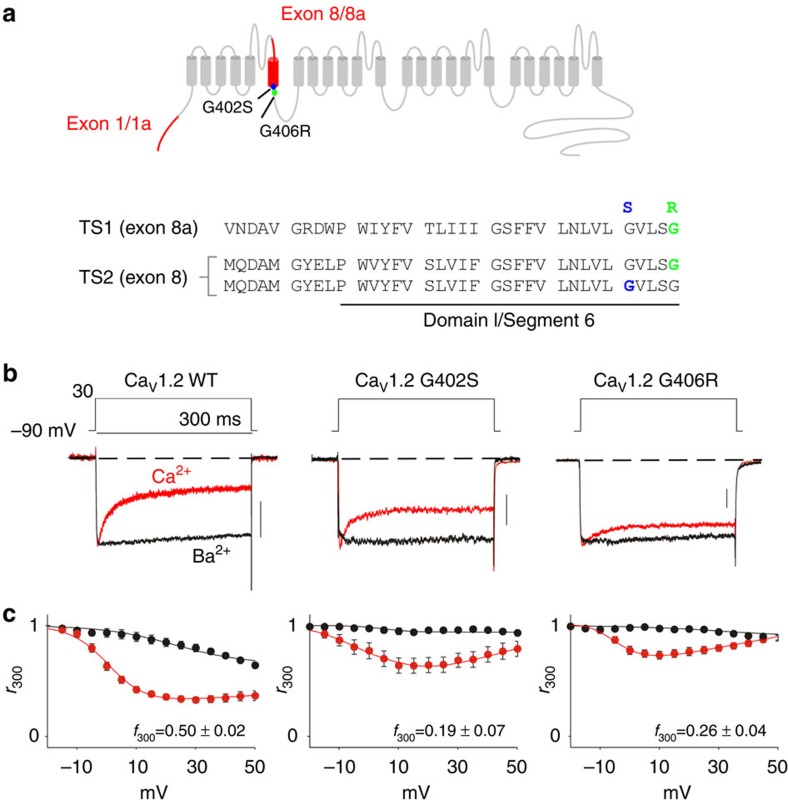
Timothy Syndrome mutations reduce CDI. (**a**) Diagram of Timothy Syndrome mutations within human Ca_V_1.2. Exon 8 is expected to be paired with exon 1a in cardiac channels. (**b**) Exemplar whole-cell current traces in Ca^2+^ (red) and Ba^2+^ (black). CDI is seen as the faster decay of the Ca^2+^ versus Ba^2+^ trace. Channels are co-expressed with β_2a_ to allow examination of CDI independent of VDI effects. In addition to their VDI effects, both Timothy Syndrome mutations confer a significant decrease in CDI (middle, right). Scale bars are 200 pA and refer to the Ca^2+^ trace. (**c**). Population data, the fraction of peak current remaining after 300-ms depolarization (*r*_300_) is plotted for Ba^2+^ and Ca^2+^ currents. The difference between Ca^2+^ and Ba^2+^ relations at 30 mV (*f*_300_) specifies the isolated effect of CDI. Error bars represent±s.e.m., *n*=10, 8 and 12 for WT, G402S and G406R, respectively.

Given the ambiguity regarding the effects of the TS mutations on the biologically relevant CDI process^11^,^20^, we first undertook careful dissection of Ca^2+^ regulatory changes caused by each TS mutation. Here, we chose conditions which minimize VDI in Ca_V_1.2, allowing isolated study of CDI. To accomplish this, we co-expressed our human Ca_V_1.2 channels with the β_2a_ auxiliary subunit, which is known to decrease VDI in Ca^2+^ channels^22^. In addition, as exons 1 and 8 are known to co-splice^3^, we constructed two naturally expressed human cardiac (exon 1a/exon 8) and smooth muscle (exon 1/exon 8a) splice variants^3^ harbouring the TS mutations. We could now use whole-cell patch-clamp recordings to measure CDI (Fig. 1b), which can be seen as the faster decay of the Ca^2+^ current (red) as compared with the Ba^2+^ current (black) and is quantified as the ratio of Ca^2+^ current remaining after 300 ms (*r*_300_)^23^. Under these conditions, both G402S and G406R channels exhibited a striking reduction of CDI (Fig. 1) as compared with wild-type (WT) Ca_V_1.2 channels. Further, this reduction in CDI was clearly present in either of the physiologically relevant splice backgrounds (Supplementary Fig. 2). We thus demonstrated a strong effect of either TS mutation on CDI in Ca_V_1.2 channels. In fact, even in the context of strong VDI seen in the presence of the β_1b_ subunit, the effects on CDI are readily apparent in both splice backgrounds (Supplementary Figs 3 and 4) and can be detected in adult myocytes (Supplementary Fig. 5). In all, these data firmly establish a clear effect on CDI by either of the two TS mutations across multiple experimental conditions.

### An allosteric model predicts opposing mechanisms of CDI loss

**Figure 2:**
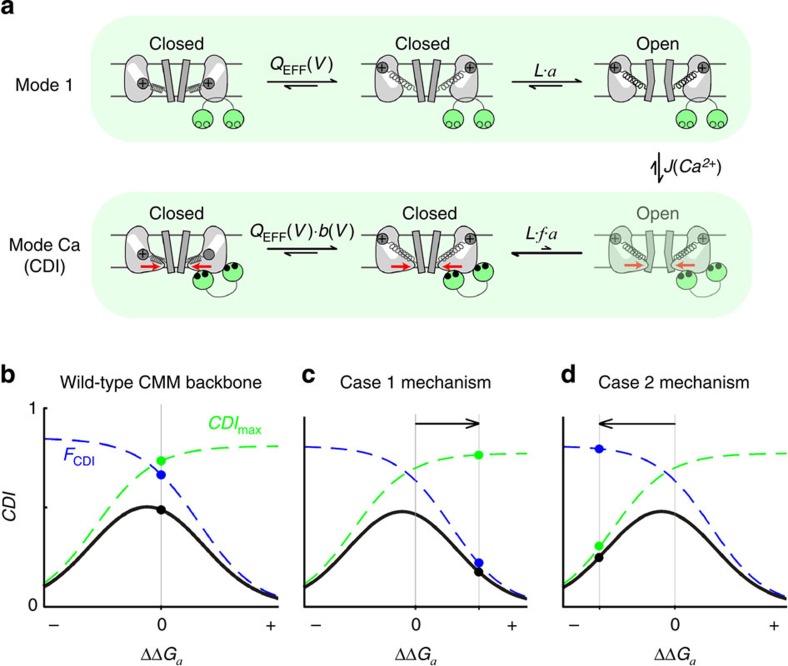
An allosteric model could explain a decrease in CDI. (**a**) An allosteric CDI mechanism^9^,^24^. Ca^2+^ (black circles) binding to CaM (green) drives channels into mode Ca, which exhibits lower open probability (*P*_O_). (**b**) The model predicts two main components of CDI. *F*_CDI_ (blue) is the fraction of channels in mode Ca (dependent on *J*(Ca^2+^) and thus *P*_O_) and *CDI*_*max*_ (green) results from the relative ability of the channel to open once in mode Ca. The combination of these two curves defines the total CDI of a channel (black). (**c**) Mutations which inhibit channel opening (ΔΔ*G*_*a*_>0) decrease Ca^2+^ entry, reducing *F*_CDI_ (blue). These channels will exhibit lower CDI (black) due to a decreased ability to populate mode Ca. (**d**) Alternatively, enhanced channel opening (ΔΔ*G*_*a*_<0), increases openings in mode Ca, thereby decreasing *CDI*_max_ (green) and overall CDI (black).

We next turned to the underlying mechanisms of CDI disruption. We began by considering an allosteric mechanism of CDI known to underlie gating of Ca_V_1.2 and Ca_V_1.3 channels^9^,^10^,^24^. According to this model (Fig. 2a), in the absence of CDI, channel gating is described by a ‘mode 1' scheme (top row), where *Q*_EFF_(*V*) is the voltage-dependent equilibrium constant of the channel voltage sensors, *L* governs the voltage-independent final concerted opening step of the channel and *a* is a scaling factor representing the effects of the TS mutations on channel activation (*a*=1 for WT channels). Upon opening, Ca^2+^ enters and binds to CaM (black circles), ultimately driving the channel into the ‘mode Ca' regime (bottom row). This mode is characterized by a lower open probability that results from a decreased equilibrium constant between the last closed state and the open state. In particular, the magnitude of decrease is specified by factor *f*. According to this allosteric mechanism, CDI can be decomposed into two components. The first is specified by the metric *F*_CDI_, which quantifies the fraction of channels that transition from mode 1 to mode Ca in the steady state. As expected, *F*_CDI_ depends upon *J*(Ca^2+^). The second component is quantified by *CDI*_max_, the CDI that would be observed if all of the channels were to transition from mode 1 to mode Ca. Accordingly, *CDI*_max_ reflects the reduction in open probability of mode Ca relative to mode 1. The combination of these two components thereby defines the total CDI that results at steady state, where CDI=*CDI*_max_ × *F*_CDI_ (refs 9, 10).

Given this framework, we could conceptualize the potential effects of the TS mutations. Since these mutations are situated near the intracellular channel activation gates^25^, one might expect changes in the ease of transitions from closed to open states, as represented by the scaling factor *a* applied to the final opening step^9^,^10^. By representing this scaling factor in terms of the change in energy required for the channel to open (ΔΔ*G*_*a*_=*RT·*log(*a*)), simulations of the model in Fig. 2a suggest that reductions in CDI could actually arise in two distinct ways. First, mutations that make opening more difficult (*a*<1, ΔΔ*G*_*a*_>0) would decrease Ca^2+^ influx, attenuate *J*(Ca^2+^) and thus reduce *F*_CDI_ (Fig. 2b, blue). In this way, channels would exhibit lower CDI due to decreased occupancy of mode Ca at steady state (case 1). Alternatively, if the channel becomes easier to open (*a*>1, ΔΔ*G*_*a*_<0), then the open probability in mode Ca would also increase, resulting in decreased *CDI*_max_ (Fig. 2b, green). In this scenario, CDI decreases despite entry into mode Ca, because of an attenuated decrement in open probability upon entering mode Ca from mode 1 (case 2). Thus, either a decrease or an increase of energy required for the final opening step may reduce CDI, but with an important predicted difference. For case 1, the *F*_CDI_ reduction is primarily due to decreased Ca^2+^ influx and too little Ca^2+^ driving entry into mode Ca. As such, CDI could be restored if sufficient Ca^2+^ were available. Case 2, on the other hand, is the result of a reduction in *CDI*_max_ which cannot be overcome by increasing the Ca^2+^ that triggers inactivation.

**Figure 3:**
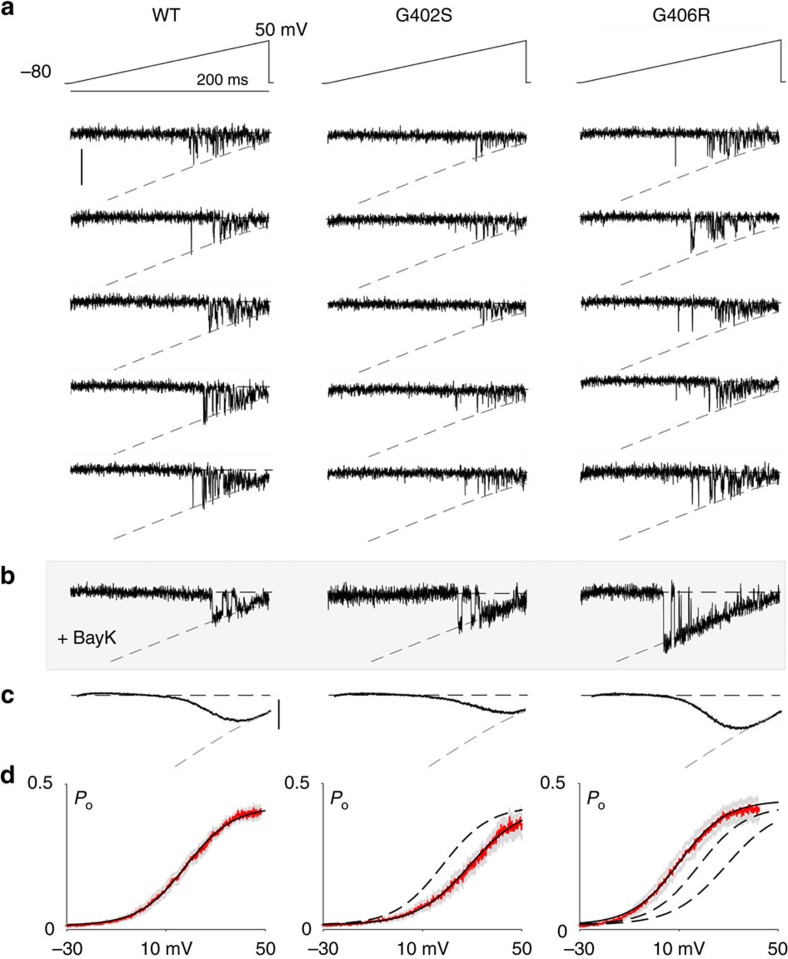
Opposing shifts in channel activation in TS channels. (**a**) Exemplar on-cell current recordings in response to a voltage ramp (top). Channel openings are fit by the GHK equation (grey dashed line). Scale bar, 1 pA. (**b**) Addition of Bay K 8644 increases mode 2 openings, verifying channel number and GHK fit. (**c**) Current averaged over multiple patches gives the current voltage relation (black). The grey dashed relation is now the GHK relation scaled down by *P*_O/max_. Scale bar, 0.2 pA. (**d**) *P*_O_ as a function of voltage, averaged across cells (red) with Boltzmann fit (black). Grey displays ±s.e.m. G402S (middle) is clearly right shifted (case 1) compared with WT displayed as dashed line for comparison. G406R (right) shifts left (case 2) as compared with WT and G402S (dashed curves). *n*=13, 8 and 7 for WT, G402S and G406R, respectively.

To explore whether TS mutations might actualize one or both of these model scenarios, we scrutinized mutant TS channels for changes in channel activation. To this end, we utilized single-channel recordings to determine the open probability (*P*_O_) of the channels as a function of voltage (Fig. 3). Here, voltage ramps were applied to single Ca_V_1.2 channel with Ba^2+^ as the charge carrier. Initially, gating corresponds to typical Ca_V_1.2 mode 1 behaviour (Fig. 2a)^24^, while the addition of Bay K 8644 resulted in a substantial increase in mode 2 gating in both TS mutants (Fig. 3b) as expected for Ca_V_1.2 (ref. 26). This increased channel opening permitted clear resolution of the single-channel conductance (dashed grey line; *g*=0.018 pA mV^−1^), and significantly aided in determining the number of channels in each patch, required for accurate calculation of *P*_O_. We could now divide the ensemble average current into the fully open current level, to get the *P*_O_–*V* relationship. We find that WT and both TS mutant channels share a maximal *P*_*O*_ of 0.4 at saturating depolarization (Fig. 3d). On the other hand, the *V*_½_ of each construct is markedly different from the WT. The G402S mutation confers an ∼10-mV depolarizing shift in activation (Fig. 3d, middle; Supplementary Fig. 6), consistent with case 1 of our model (ΔΔ*G*_*a*_>0). In contrast, the G406R channels exhibit an opposite, ∼10 mV, left shift of *P*_*O*_–*V* curves (Fig. 3d, right; Supplementary Fig. 6), fitting with a case 2 profile (ΔΔ*G*_*a*_<0). This dichotomy of effects raised the intriguing possibility that the two TS mutations in fact elaborate opposing mechanisms of CDI loss, despite superficial similarities in their inactivation defects. Contrasts in the actual underlying mechanism of action might then explain differences in disease progression among patients, and point the way to improved therapies customized to the individual challenges of each scenario.

### Direct evidence for opposing mechanisms of CDI loss

Though compelling, the contrasting *P*_O_–*V* relations for WT and TS channels in themselves do not substantiate the existence of case 1 and/or case 2 mechanisms. Direct tests of such mechanisms could be undertaken if we could control the Ca^2+^ signal driving CDI, in a manner independent of variable Ca^2+^ fluxing through channels with differing activation gating properties. This capability would allow for direct control over *F*_CDI_ (a function of Ca^2+^), while leaving *CDI*_max_ (a function of channel gating) unaltered. To this end, we employed Ca^2+^ photo-uncaging to manipulate the intracellular Ca^2+^ concentration. In addition, we used the monovalent permeant ion Li^+^ as the inward charge carrier through channels, so as to ensure that uncaged Ca^2+^ was the sole source of Ca^2+^. However, this configuration of channel permeation heightens the chance of pore block at the channel selectivity filter by uncaged Ca^2+^ (Supplementary Fig. 7)^27^,^28^,^29^; such blockade would complicate resolution of CDI apart from block. To reduce this effect, we utilized a known pore mutation (E736A) which reduces the selectivity of the channel for Ca^2+^ versus Li^+^ (ref. 29), thus allowing for resolution of CDI in relative isolation (Supplementary Fig. 7).

**Figure 4:**
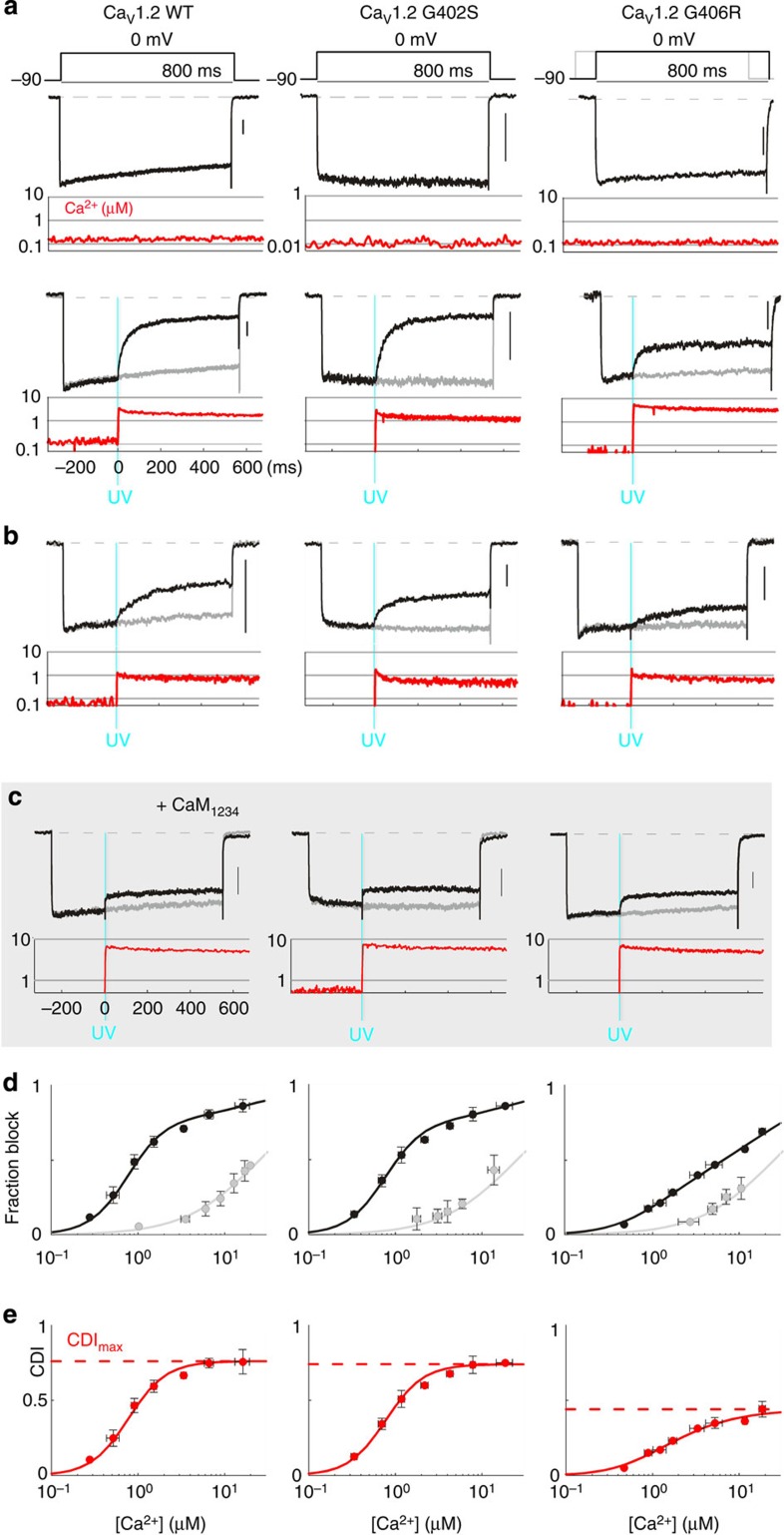
Controlling the Ca^2+^ input reveals opposing mechanisms behind TS variants. (**a**) Ca^2+^ uncaging in Ca_V_1.2 (E736A)^27^. Channels exhibit little inactivation with lithium as the charge carrier (black). Alexa568 and Fluo-4FF calcium dyes were dialyzed into cells, allowing simultaneous Ca^2+^ measurement (red). Internal Ca^2+^ was caged with DMNP-EDTA, allowing a step release of Ca^2+^ upon UV flash (cyan). Below, CDI is seen as a strong decrease in current (black) compared with pre-flash current (grey). Scale bar, 200 pA. (**b**) Lower levels of Ca^2+^ were achieved by varying the Ca^2+^ cage and UV flash intensity. (**c**) UV flash data obtained with CaM_1234_ yields the extent of residual Ca^2+^ pore block. (**d**) Fraction block produced by UV flash as a function of Ca^2+^ concentration for Ca_V_1.2/CaM (black). Grey relation shows the residual pore block from CaM_1234_ experiments. Error bars indicate±s.e.m. (**e**) The true CDI curve corrected for residual pore block. The curve for G402S (middle) is remarkably similar to WT Ca_V_1.2 (left), resulting in a nearly identical *CDI*_max_ (red dashed line) while the *CDI*_max_ of G406R is significantly reduced (right panel, red dashed line).

Thus armed, we undertook Ca^2+^ photo-uncaging of TS channels. DM nitrophen (DMNP-EDTA) provided an appropriate Ca^2+^ cage, supporting near step release of Ca^2+^ upon brief ∼1 ms flashes of ultraviolet (UV) excitation (measured by Ca^2+^ imaging with Fluo-4FF). Combined with patch-clamp electrophysiology, Ca^2+^ uncaging provided a controlled Ca^2+^ input and a clear readout of CDI. As before, Ca_V_1.2 channels displayed little inactivation when internal Ca^2+^ was kept low (Fig. 4a, top). When Ca^2+^ was uncaged during the depolarizing step, CDI manifested as a prominent decay in current (Fig. 4a, bottom). By measuring this CDI at various Ca^2+^ levels (Fig. 4b), robust definition of a Ca^2+^ response curve for Ca_V_1.2/CaM could be achieved by plotting the extent of inhibition versus the Ca^2+^ concentration (Fig. 4d). For this curve to be fully reflective of CDI alone, any residual pore block must first be accounted for. To this end, we utilized our Ca^2+^ uncaging protocol on the same channels, but co-expressed with a mutant CaM (CaM_1234_) which lacks Ca^2+^ binding (Fig. 4c). In this configuration, channels could not undergo CDI and we could independently measure the remaining pore block of the channel. We found that Ca^2+^ pore block had a *K*_d_ of 24 μM, and a Hill coefficient of unity, regardless the type of channel (Fig. 4c,d), an outcome consistent with the original description of the E736A mutation^27^,^29^. We could now divide the residual pore block relation into our uncaging data, thus explicitly resolving the Ca^2+^ response curve for CDI in the Ca_V_1.2/CaM complex (Fig. 4e).

As expected from whole-cell recordings, Ca_V_1.2 channels exhibit a large *CDI*_max_ (0.77), with a *K*_d_ of 0.78 μM and a Hill coefficient of 2 (Fig. 4e). Turning to the G402S mutation, case 1 predicts that the *F*_CDI_ deficit of these channels should be rectified by a sufficiently large Ca^2+^ input. Indeed, G402S channels exhibited pronounced CDI in response to a large Ca^2+^ step (Fig. 4a,b, middle) resulting in a *CDI*_max_ comparable to WT. This outcome directly establishes a case 1 scenario. G406R, on the other hand, fails to exhibit enhanced CDI, even in the presence of a saturating Ca^2+^ signal (Fig. 4a,b, right). Indeed, response curve analysis of this mutant channel revealed a considerable reduction in *CDI*_max_, directly substantiating a case 2 scenario (Fig. 4e, right). Thus, we have validated two opposing mechanisms underlying TS CDI deficits: G402S causes a decrease in *F*_CDI_, while G406R primarily results in a reduction of *CDI*_max_.

### Implications for pathogenesis

**Figure 5:**
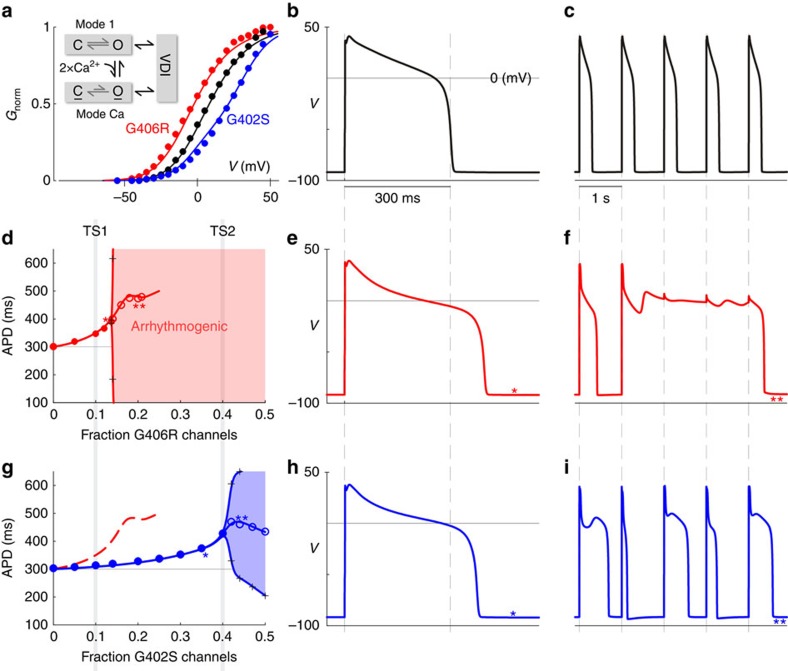
Effects of opposing mechanisms on modelled action potentials. (**a**) The LRd model was used to gauge TS mutation effects on cardiac ventricular APs. Model activation curves (black curve) fit well the tail-current data (black circles). G406R (red) and G402S (blue) curves shifted to match experiments (circles). Inactivation parameters were similarly adjusted. (**b**) A single WT AP generated by the model. (**c**) Modelled WT APs displayed on an expanded timebase during 1-Hz pacing to show stability. (**d**) Severe effects of G406R channels. Variable G406R expression modelled by including a variable fraction of mutant versus WT channels. At first, APD increased monotonically, then become patently unstable before reaching TS2 levels. (**e**) Significant APD prolongation at ∼15% G406R. Asterisk indicates corresponding data in **d**. (**f**) Profound AP prolongation due to arrhythmogenic early after depolarizations at ∼20% G406R. Double asterisk indicates corresponding data point in **d**. (**g**) Milder G402S mutation (blue) effects. Significant APD prolongation does not arise until mutant channels reach the anticipated mean of TS2 patients. The G406R curve is reproduced for comparison (dashed red). (**h**) APD prolongation at ∼40% G402S. Asterisk indicates corresponding data in **g**. (**i**) Slight further increase of G402S (∼44%) induces alternans. Double asterisk indicates corresponding data point in **g**.

Given the clear contrast between the biophysical mechanisms underlying the two TS mutations, we explored potential differences in pathogenesis. As a first step, we focused on the cardiac effects of TS, utilizing a widely employed ventricular myocyte model, the Luo–Rudy (LRd) model^30^. This *in silico* model, formulated for guinea pig, was here customized to incorporate L-type channel parameters constrained by our experimental measurements, and to more closely approximate the baseline behaviour of the human heart. In particular, the tail-current activation curves, obtained for each channel in this study (Supplementary Fig. 6), were used to constrain the simulated L-type channel activation properties as shown in Fig. 5a. Here, the solid lines represent model fits, and circles reproduce experimental data shifted to account for surface charge effects. Additionally, inactivation parameters for both VDI and CDI were adjusted to correspond to data from this study. Finally, we explicitly simulated two pools of L-type channels, to account for the heterozygous nature of TS mutations. Because TS1 and TS2 differ primarily in their expression levels in the heart, we allowed the fraction of TS-affected channels to be adjusted in the model. With appropriate customizations of the LRd model in hand (see Supplementary Note for details), we looked first at the APs generated with the WT Ca_V_1.2 channels at steady state. Here, in response to 1 Hz pacing, we obtained an APD of 300 ms (Fig. 5b), and a slower timebase displays that the repetitive electrical responses of the model are stable, and simulated AP waveforms closely resemble those observed in humans (Fig. 5c)^31^. We could now consider the effects of variable expression of TS channels in the model.

For G406R channels, which exhibit both enhanced activation gating and heightened opening in mode Ca, the effects were particularly severe. Upon initial increase of the fraction of G406R channels, the APD prolonged monotonically (Fig. 5d,e). However, upon further increase, we encountered a bifurcation of the APD in the model, where electrical responses became patently unstable (Fig. 5d,f). Interestingly, this region of beat to beat APD instability was achieved at levels just higher than those expected for TS1, and significant arrhythmogenesis would predominate at the higher levels of mutant channel expression anticipated for TS2 patients. Specifically, significant APD prolongation was seen at about 10% G406R expression, roughly corresponding to TS1 (heterozygous expression within exon 8a), while APD instability was seen by 20%.

By contrast, the effects of the G402S mutation, with its decreased propensity to open via activation gating and normal *CDI*_max_, appear to be substantially milder (Fig. 5g–i). Substantial APD prolongation does not arise until the fraction of mutant channels reaches the anticipated mean level of TS2 patients (∼40% G402S versus WT as expected from a heterozygous mutation in exon 8). Interestingly, for TS2 patients, a slight increase in the fraction of G402S channels beyond the mean anticipated TS2 level is predicted to induce outright arrhythmia (at ∼44%). Again, TS2 patients with the milder G402S mutation are nonetheless perched on the threshold of cardiac instability.

### Direct test of model results in adult myocytes

**Figure 6:**
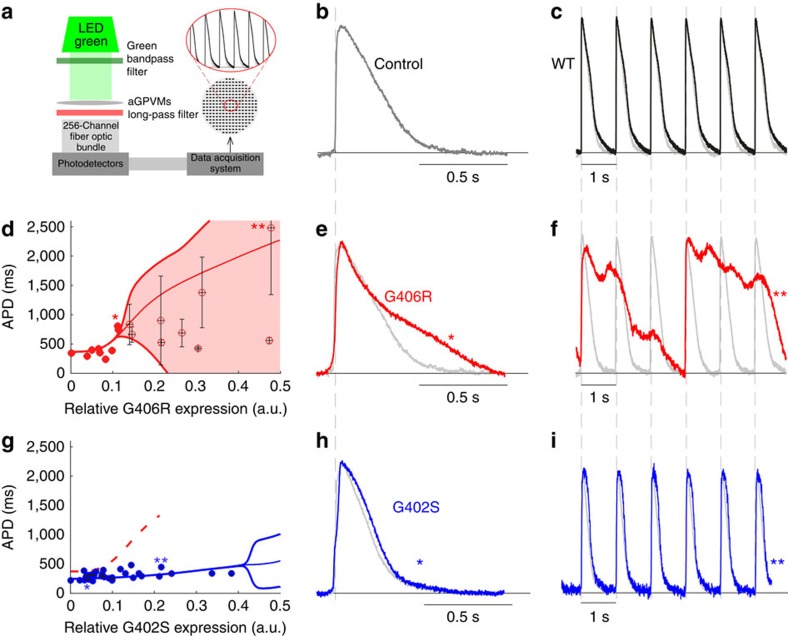
Verification of nonlinear threshold behavior in aGPVMs. (**a**) aGPVM APs were recorded via a standard optical mapping setup, using the voltage-sensitive fluorescent dye di-4-ANEPPS. (**b**) Average AP recorded from uninfected aGPVMs. (**c**) Lentiviral-mediated expression of recombinant WT Ca_V_1.2 channels caused only slight prolongation of the cardiac APs (black trace) compared with control cells (grey, as in **b**). (**d**) Variable G406R expression was determined by GFP expression. As in the LRd model, APs (measured at 80% repolarization) initially increased monotonically without appreciable variability (data plotted as mean±s.d.). Upon slight further increase in G406R channels, APs became flagrantly unstable as cells moved past a bifurcation point into a highly arrythmogenic region. (**e**) Before the bifurcation region, G406R (red) causes significant APD prolongation as compared with control (grey). Asterisk indicates corresponding data point in **d**. (**f**) Outright arrhythmia at higher expression levels (red) confirms the LRd model. Double asterisk indicates corresponding data point in **d**. (**g**) Significant APD prolongation by G402S (blue) does not arise, even past expression levels corresponding to a bifurcation point for G406R channels (red dashed curve reproduced from **d**). (**h**) Low G402S expression (blue) causes only a slight change in action potential shape compared with control (grey). (**i**) Higher G402S expression fails to induce severe irregularity of responses, confirming the ability of myocytes to withstand a higher load of G402S channels.

The bifurcation phenomena identified by the LdR model hold important implications for pathogenesis and potential therapy. To test this prediction of the model more directly, we expressed WT, G406R and G402S channels in cultured monolayers of adult guinea pig ventricular myocytes (aGPVMs), a preparation exhibiting APs with APDs comparable to human, making them suitable for exploring long QT phenomena^32^. Recombinant channels were fused to GFP, allowing fluorescence measurements to report approximate expression levels. In this system, lentiviral-mediated expression of recombinant WT Ca_V_1.2 channels caused only a slight prolongation of the cardiac APs compared with control cells (Fig. 6c). This lack of AP prolongation due to overexpression is consistent with previous reports that these myocytes may lower endogenous channel expression in response to exogenous expression, thus keeping the total number of channels relatively constant^33^. This compensatory mechanism enables direct comparison of the aGPVM experiments with the model predictions.

Unlike WT channels, variable G406R expression (as quantified by GFP intensity), produced a marked effect on the APs. As in the LRd model, APs initially increased monotonically without appreciable variability (Fig. 6d,e). Upon slight further increase in G406R channels, however, APs became flagrantly unstable, as cells moved past a bifurcation point into a region of frank electrical instability (Fig. 6d,f). Hence, the instability threshold seen in the LRd model is confirmed in mammalian cardiac myocytes. Furthermore, as in our simulations, the G402S mutation produced a less pronounced APD prolonging effect. While low levels of expression did cause a slight increase in APD, significant APD prolongation by G402S (blue) did not arise, even past expression levels corresponding to a bifurcation point for G406R channels (Fig. 6g–i), confirming the ability of myocytes to withstand a higher load of G402S channels.

## Discussion

CDI represents a vital feedback mechanism required for proper Ca^2+^ signalling in cardiac, neuronal and immune systems. Disruption of CDI in ventricular myocytes has been shown to cause substantial AP prolongation^16^ and similar defects in patients have now been discovered underlying a severe form of long QT syndrome^19^. Yet the effects of the TS mutations on this vital form of channel regulation have remained unclear^11^,^20^. Here, we have uncovered a significant effect of both TS mutations on CDI, and have further demonstrated a remarkable divergence in the mechanisms underlying this loss of CDI. Such opposing mechanisms leading to the prolongation of the cardiac APs stand in contrast to the view that these mutations produce parallel defects in channel gating (primarily VDI), and thus lead to similar manifestations in patients^1^,^34^,^35^,^36^. In fact, some of the variations in symptoms among TS patients could be explained by the different CDI deficiencies of the two mutations. While these CDI deficits alone may not account for the full spectrum of TS symptoms^12^,^13^,^14^,^15^, they are likely a major contributing factor underlying the life-threatening arrhythmias seen in TS patients. Overall, the mechanistic dichotomy of CDI effects indicates a need to tailor the treatment of TS to each genetic variant.

The distinct effects of the TS mutations on channel gating give rise to a nonlinear dependence on TS gene expression. Importantly, the divergent mechanisms underlying the two TS mutations results in a significant difference in the amount of TS channel expression required to reach a threshold for the induction of arrhythmia. Given the implications of this nonlinear threshold behaviour, we replicated our *in silico* results in cultured monolayers of aGPVMs. The nonlinear threshold behaviour observed in the model is remarkably consistent with the behaviour observed in aGPVMs, confirming that the model predictions extend to networks of native myocytes. While our results predict that TS patients may reside on the cusp of an area of electrical instability, the exact location of this bifurcation point will depend on heart rate, phosphorylation state of the channels and other variations in baseline cardiac parameters. As such, even natural variations in cardiac function may shift the threshold for electrical instability such that patients are at high risk for severe arrhythmia. However, this study also offers new hope for patients. The nonlinear dependence of the APD on channel expression implies that it may not be necessary to block or correct all the mutant channels. Rather, significant improvement may occur with only a small shift in the complement of mutant versus WT channels, either by specific pharmacological blockade or an alteration in channel expression.

The *in silico* and live cell data presented in this study illustrate that the severity of TS is a function of both the specific mutation (G406R versus G402S) and the alternate exon in which it is expressed (exon 8 versus exon 8a). This dependence on gene expression and mutation type correlates well with the severity of symptoms in TS patients. In the heart, the lower expression of the TS mutation in TS1 leads to a milder phenotype and increased patient survival^2^,^37^,^38^,^39^,^40^. For G406R, this level of expression positions TS1 patients near an area of electrical instability. However, for patients harbouring the G402S mutation, the lower expression level of TS1 may produce such a minimal phenotype that they may not exhibit overt symptoms, consistent with the lack of reported TS1 G402S patients. However, this potential population of patients should not be ignored, as it is possible that the more subtle effects on APD could put them at higher risk for acquired LQTS. For TS2, on the other hand, expression levels in patients are expected to be four times that of TS1 patients^1^,^2^,^3^. For G406R, this level of expression would be expected to produce enormously increased QT durations and APD instability which would conspire to be lethal in most cases. This may rationalize the exceedingly small population of diagnosed G406R mutations in TS2 patients^1^. It appears likely that strong compensatory mechanisms must arise in these TS2 patients during development to allow them to withstand the severe effects of the G406R mutation. For G402S, however, the higher TS2 gene expression levels again position patients near a threshold for electrical instability^1^,^5^,^34^,^41^,^42^. Further, recent case reports have identified several patients with mosaic expression of a TS mutation^38^,^39^,^40^. These patients are often identified among the healthy parents of TS children, indicating that the lower gene dosage induced by mosaic expression of the mutant gene may prevent outright cardiac symptoms, consistent with the predictions of this study.

**Figure 7:**
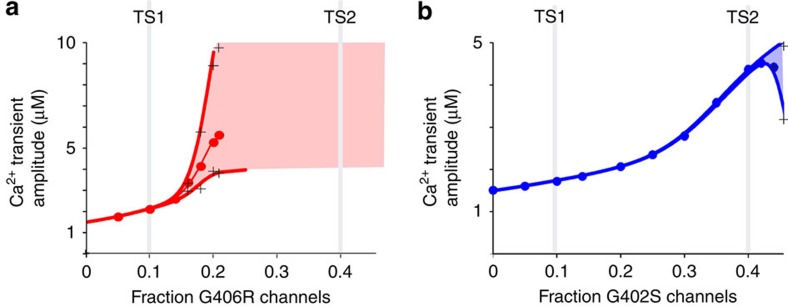
TS effects on Ca^2+^ transient amplitudes. (**a**) The LRd model was used to gauge TS mutation effects on Ca^2+^ transient amplitudes. Data corresponds to the same simulations run in Fig. 5. Increasing the fraction of G406R channels increased the average Ca^2+^ transient amplitude (red circles). (+) indicates minimum and maximum amplitudes for an individual simulation. (**b**) Increasing the fraction of G402S channels also increased the average Ca^2+^ transient amplitude (blue circles) but with a shallower dependence on the fraction of channels.

In addition, these TS effects on APD may be accompanied by significant effects on EC coupling. In particular, the ventricular myocyte model predicts that both mutations cause a gene-dose-dependent increase in the amplitude of the Ca^2+^ transient (Fig. 7). This effect is consistent with previous studies of the G406R mutation^43^,^44^,^45^ and is indicative of an increased SR Ca^2+^ load in TS myocytes^45^. Our results support the hypothesis that the TS channels produce an increase in the EC coupling gain^45^. Importantly, this effect holds not only for the G406R channel, which exhibits a left shift in channel activation, but for the right-shifted G402S channel as well (Fig. 7), illustrating the critical role of Ca^2+^ channel inactivation in EC coupling^45^. Moreover, the altered Ca^2+^ load due to TS channels may actually alter the function of the WT Ca_V_1.2 channels within the cell. Specifically, a larger SR Ca^2+^ release may increase the kinetics of CDI for WT Ca_V_1.2 channels, while the lack of CDI in the mutant channels would tend to increase the steady state Ca^2+^ current. This mixed population effect may explain why myocytes from TS mice have an overall increased rate of inactivation and a concurrent decrease in steady state inactivation^45^. In all, gating defects of the TS channels appear to disrupt both the electrical properties and Ca^2+^ handling within myocytes, leading to significant arrhythmogenesis.

Beyond these initial TS channels, the number of mutations identified in Ca_V_1.2 is fast increasing. Many of these mutations produce symptoms which are quite similar to TS^46^,^47^,^48^, while others lack a number of the TS features^48^,^49^,^50^. This may be partially explained by the differing locations of these mutations. While many lie in similar S6 channel regions as the TS mutations, several have been identified in the N or C tail^48^,^49^,^51^,^52^ of the channel, as well as the II-III or I-II loop^49^,^50^. Nonetheless, the effects of these mutations on CDI has been considered in only a few of these new cases^46^,^49^. As the alteration of CDI may be a common feature of many Ca^2+^ channelopathies, this may represent an important avenue of further investigation. As none of the recently reported Ca_V_1.2 mutations are expressed in an exon known to undergo alternative splicing, it is possible that the particular biophysical defects of these channels may be tolerated at a higher level as compared with the TS mutations. Thus, studies such as this one, which span from single molecule biophysics to network level phenomenon, may enable a deeper understanding of the mechanisms underlying these channelopathies and provide insight into novel treatments for these patients.

More broadly, this nonlinear threshold phenomenon is likely not limited to TS. Indeed, there is reason to wonder whether channelopathies that most frequently attract clinical surveillance are those that poise patients on the threshold of outright instability. Channel mutations that position patients further from a domain of overt dysfunction may never garner clinical observation, while mutations that consign individuals to persistent and severe arrhythmias may curtail survival. It is therefore likely that the lessons learned from TS will shed new direction on a multitude of channelopathies.

## Methods

### Molecular biology

The human α_1C_ cardiac isoform was a kind gift from Tuck Wah Soong^53^. A topo clone was generated spanning the front end of the channel from HindIII to ClaI and the G406R and G402S mutations were introduced by quick change ligation (primer for G402S: CGTTCTAAATCTGGTTCTCAGTGTGTTGAGCGGAGAGTTTTCCAAAG , primer for G406R: CGTTCTAAATCTGGTTCTCGGTGTGTTGAGCAGAGAGTTTTCCAAAG ). The construct was then transferred via HindIII and ClaI into the full human α_1C_ backbone in pcDNA3 (Acc# Z34810). For lentivirus expression, a GFP was attached to the C terminus of these channels at amino acid 1671 via introduction of an xba site at this location. The channels were then subcloned into the lentiviral plasmid, pRRLsin18.cPPT.CMV.eGFP.Wpre^54^ which was a generous gift from Dr Gordon Tomaselli. Lentivirus production and purification were then carried out in Hek293T cells using a third generation lentiviral expression system^54^.

All flash experiments utilized a pore mutant which reduced Ca^2+^ pore block when patching in 80 mM Li^+^ external solution^27^,^55^. An E736A permeation mutant was introduced by overlap extension PCR on WT Ca_V_1.2. The E736A mutated fragment was then ligated into each channel construct using SrgAI and PpuMI. All sequences subject to PCR were verified by sequencing.

### Electrophysiology

HEK293 cells (ATCC) were transfected using calcium phosphate^56^. Cells were co-transfected with 8 μg of rat brain β_1B_ or β_2a_ (ref. 57), 8 μg of rat brain α_2δ_ (ref. 58), and 1–8 μg of Ca^2+^ channel α_1_ subunit and 2 μg of SV40 T-antigen; 8 μg of cDNA for rat brain CaM_1234_ was added as required. A flourophore was used in all transfections for identification of transfected cells. For standard whole-cell experiments, the beta subunit was contained in a GFPIR vector, while for Flash experiments, CFP was added to the transfection. CaM_1234_ was contained within a CFPIR vector so that cells with high levels of CaM_1234_ could be distinguished. Room temperature, whole-cell recordings were performed 1–2 days after transfection, using Axopatch 200 A/B amplifiers (Axon Instruments). P/8 leak subtraction was used, with series resistances of 1–2 MΩ after >70% compensation. Currents were filtered at 2 kHz (4-pole Bessel), and sampled at 10 kHz. For whole-cell experiments, internal solutions contained, (in mM): CsMeSO_3_, 114; CsCl_2_, 5; MgCl_2_, 1; MgATP, 4; HEPES (pH 7.4), 10; and BAPTA, 10; at 295 mOsm adjusted with CsMeSO_3_. External solutions contained (in mM): tetraethylammonium-MeSO_3_, 140; HEPES (pH 7.4), 10; and CaCl_2_ or BaCl_2_, 40; at 300 mOsm, adjusted with TEA-MeSO_3_.

For Flash experiments, the internal solution contained, (in mM): CsMeSO_3_, 135; CsCl_2_, 5; HEPES (pH 7.4), 40; Flou4FF, 0.01; and Alexa568, 0.0025. In addition, DMNP-EDTA, CaCl_2_ and citrate were added such that baseline Ca^2+^ levels were below 100 nM and varying Ca^2+^ steps could be achieved. The range of concentrations used were, (in mM): DMNP-EDTA, 1–4; citrate, 1–20; CaCl_2_ 0.3–3.4 (adjusted so as to be 60–80% of the DMNP concentration). For Flash experiments, external solutions contained (in mM): TEA-MeSO_3_, 70; HEPES (pH 7.4), 10; and LiCl, 80. Data were analysed by custom MATLAB software (Mathworks, MA, USA), with average data shown as mean±s.e.m. Ca^2+^ was calculated for each cell based on measured *R*_min_ (40 mM EGTA) and *R*_max_ (1 mM EGTA, 3 mM Free Ca^2+^ adjusted with CaCl_2_ corresponding to the DMNP-EDTA concentration in the internal solution). All calibration solutions were made in the same base internal solution as above. Ca^2+^ concentration was calculated as:


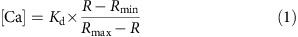


where *K*_d_ was determined to be 20 μM and *R* was the measured ratio of Fluo-4FF to Alexa568.

### Single-channel recordings

On-cell recordings were obtained at room temperature (integrating mode, Axopatch 200B; Axon Instruments). Patch pipettes (3–10 MΩ) were pulled from ultra-thick-walled borosilicate glass (BF200-116-10, Sutter Instruments), and coated with Sylgard. Currents were filtered at 2 kHz. To zero membrane potential, the bath contained (in mM): K glutamate, 132; KCl, 5; NaCl, 5; MgCl, 3; EGTA, 2; glucose, 10; and HEPES (pH 7.4), 20 at 300 mOsm adjusted with glucose. The pipette solution was the same as the Ba^2+^ bath solution in whole-cell experiments.

For ramp experiments, the voltage was ramped from −80 to 70 mV over the duration of 200 ms. The leak for each sweep was approximated using a linear fit added to an exponential fit. The unitary current relation, *i*(V), was fit to the open-channel current level using the following GHK equation^59^:


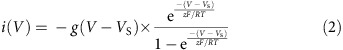


The key parameters, *g* and *zF*/*RT*, were held constant for all patches of a given construct, only allowing *V*_s_ to vary slightly from patch to patch. The leak-subtracted traces, excluding blank sweeps, were averaged together, yielding an *I*–*V* curve. The *I*–*V* curves from different patches were averaged together, compensated for the voltage shift of each patch, and the activation curve (*P*_O_ versus *V*) for each construct was determined using the GHK relation^60^.

Following acquisition of sufficient traces, BayK 8644 (Miles Inc, Pharmaceutical Div., West Haven, CT, USA) was added to the bath such that the final concentration in the bath was ∼5 μM.

### LRd model

The LRd 2007 model of the guinea pig ventricular myocyte^30^ was used to model AP effects of the TS mutations based on data collected in this study. Parameters for the L-type channel CDI, VDI and activation were adjusted to match each Ca_V_1.2 construct (Supplementary Note). Once modified, the model was run out to steady state (typically 100 pulses) with a pacing cycle of 1-Hz. Both TS and WT channels were included in the model, and the fraction of TS channels was adjusted.

### Optical mapping of aGPVMs

Guinea pig myocytes were isolated from adult guinea pigs (Hartley strain, 3–4 weeks old, weight 250–350 g) via Langendorff perfusion^32^. Myocytes were plated on laminin coated glass coverslips coated at a cell density of 10^5^ cells per ml per well. Infection of cells by lentivirus took place during the second week of culture and quantification of channel expression was done immediately before staining the cells for AP recording. Cells were cultured for a total of 3 weeks before optical mapping using 5 μM di-4-ANEPPS. During recording, cultures were paced at a frequency of 1 Hz and APs were recorded for 12 s from 253 sites using a custom built contact fluorescence imaging system^61^ which was generously made available to us by Dr Leslie Tung. Channels lacking a clear AP signal due to poor signal to noise were excluded, and the remaining AP waveforms were averaged. APD_80_ was measured from the average waveform, and s.d. was determined across the 12 s recording. To account for slight variations in different cultures, the APD_80_ for TS expressing cells was normalized to control cells from the same culture.

### Quantification of Ca_V_1.2 lentiviral expression

Lentiviral Ca_V_1.2 channels contained an attached GFP to allow quantification of relative channel expression. As aGPVMs have some intrinsic autoflouresence, true GFP expression was determined using both GFP (excitation: 470nm; emission: 530 nm) and CFP (excitation: 440 nm; emission: 480 nm) measurements as:


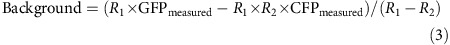



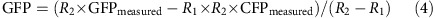



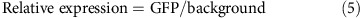


Where *R*_1_=GFP_autoflourescene_/CFP_autoflouresence_, was measured in uninfected aGPVMs each day. *R*_2_ was the ratio of GFP measured through the GFP cube compared with the CFP cube. This value was measured in HEK cells, which have minimal autoflourescence, transfected with GFP. *R*_2_ was determined to be 25 for our experimental setup. In all, this method allowed for both the removal of autofluorescence and normalization of monolayer thickness which was seen to vary between cultures.

### CDI measurements in aGPVMs

To look for effects on CDI due to the G406R mutation within a native context, we utilized acute (<48 h post isolation) aGPVMs. As these cells are resistant to most transfection protocols, we adapted a Lipofectamine transfection method described for the transfection in adult rat ventricular myocytes^4^. Briefly, acutely dissociated myocytes were plated on laminin coated coverslips for 1 h as described^5^. Once the cells had adhered to the substrate, the media was replaced with M199 media supplemented with 2 mM EGTA, 5 mM glucose, 2 mM L-Glutamine (Gibco), 5 mM HEPES, vitamin B12/penicillin (Sigma) and MEM non-essential amino acids (Gibco). After 4 h, cells were transfected with 6–12 μl Lipofectamine 2000 (Thermo Fisher) mixed in Opti-MEM (Gibco) with 3 μg Ca_V_1.2-GFP (WT or G406R) and 2 μg T-antigen. The cells were incubated overnight at 37 °C in 5% CO_2_ and rinsed with standard M199 media the next morning. Experiments were performed within 48 h of cell isolation. External solutions contained: (in mM): TEA-MeSO_3_, 140; HEPES (pH 7.4), 10; and CaCl_2_ or BaCl_2_, 5; at 300 mOsm, adjusted with TEA-MeSO_3_. Internal solution contained (in mM): Ryanodine, 0.005; CsMeSO_3_, 114; CsCl_2_, 5; MgCl_2_, 1; MgATP, 4; HEPES (pH 7.4), 10; and BAPTA, 10; at 295 mOsm adjusted with CsMeSO_3_. Note that ryanodine was added to the internal solution to view CDI in the absence of Ca^2+^ induced Ca^2+^ release^6^. Overall, this protocol resulted in a very low transfection efficiency (assessed by expression of GFP), but was sufficient to confirm an effect on CDI in primary cells.

### Voltage protocol for flash

Depolarizing voltage steps were proceeded by a P/8 leak subtraction protocol and were recorded at 5 kHz Bessel filtering. Steps were applied to each cell at 30–60 s intervals and varied between 500 and 1,000 ms in duration to allow for the currents to reach steady state before and after initiation of the UV flash. UV flash was done between 50 and 300 ms following step depolarization, where the time was adjusted to allow for the slower activating G402S channel to reach a stable current amplitude before uncaging Ca^2+^. Because analysis was done relative to the time of UV flash these time adjustments did not alter our measurements, but instead insured that all data collected corresponded with steady state conditions. In addition, the surface charge shift caused by use of Li^+^ in the external solution was accounted for by using a depolarizing step to 0 mV, which approximates (to the closest 10 mV increment) the 30 mV step done in 40 Ba^2+^ (Supplementary Fig. 8). To test this shift, experimental conditions for Ca^2+^ uncaging were approximately matched by using the same Li^+^ external solution, and 0.5 mM EDTA as an approximation of the DMNP-EDTA internal solution. The alteration in internal solutions was done simply as a practicality due to the cost of the DMNP-EDTA containing solution.

In addition to the adjustments in the amount of DMNP-EDTA Ca^2+^ cage, step size was varied by adjusting the UV flash lamp voltage within the range of 100–300 V, and by changing the capacitance of the ultraviolet lamp in the range of 500–4,000 F. Thus the amount of Ca^2+^ released from the cage could be decreased by decreasing the intensity of the UV light.

## Additional information

**How to cite this article:** Dick, I. E. *et al*. Arrhythmogenesis in Timothy Syndrome is associated with defects in Ca^2+^-dependent inactivation. *Nat. Commun.* 7:10370 doi: 10.1038/ncomms10370 (2016).

## Supplementary Material

Supplementary Information Supplementary Figures 1-8, Supplementary Note 1 and Supplementary References
