# Supplementary material for: Arrhythmogenesis in Timothy Syndrome is associated with defects in Ca2+-dependent inactivation
Source: Nat Commun. 2016 Jan 29;7:10370. doi: 10.1038/ncomms10370 (PMC4740114; doi:10.1038/ncomms10370)
Supplement: Supplementary Information — Supplementary Figures 1-8, Supplementary Note 1 and Supplementary References [file ncomms10370-s1.pdf]

Supplementary Information

Arrhythmogenesis in Timothy Syndrome is associated with defects in Ca^{2+} dependent inactivation

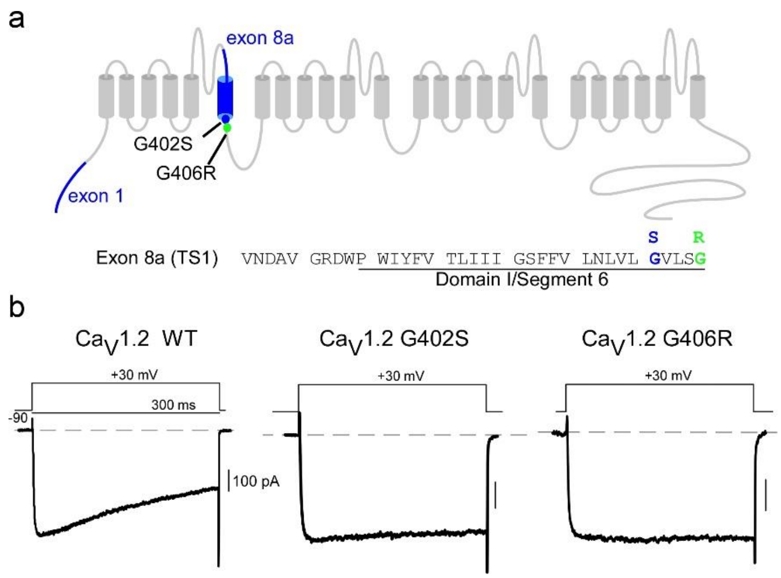

Supplementary Figure 1 | Verification of previously reported VDI deficit. (a) Diagram of Timothy Syndrome (TS) mutations within human $Ca_V1.2$. TS results from either a G406R mutation or, less commonly, a G402S mutation within the IS6 region of the channel. (**b)** Exemplar whole cell current traces in Ba^{2+} demonstrate that VDI is significantly decreased by both TS mutations (middle, right). Channels are co-expressed with β_{1b} to facilitate VDI.

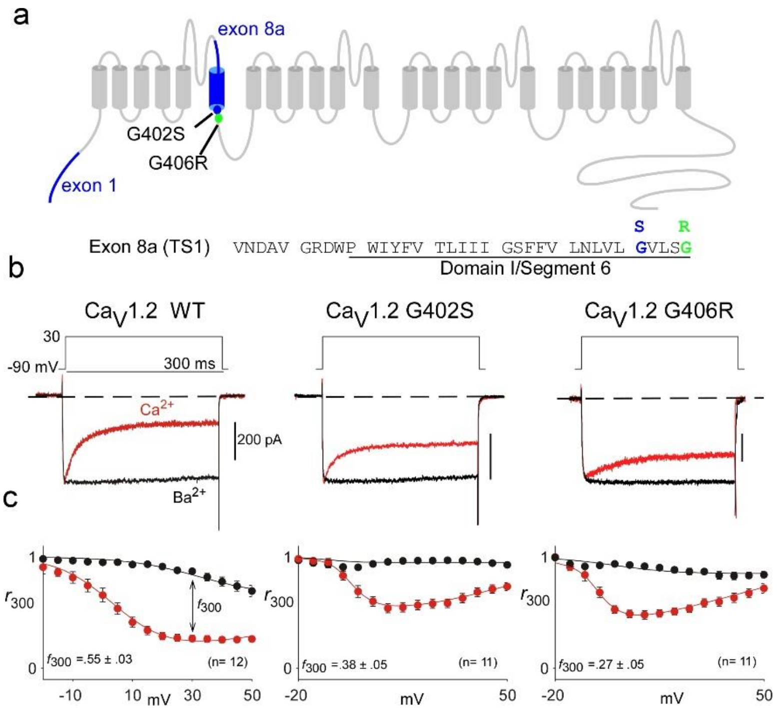

Supplementary Figure 2 | Conservation of CDI effect across channel variants. (a) Diagram of TS type 1 mutations within human $Ca_V1.2$. Exon 8a is expected to be paired with exon 1 in cardiac channels[1, 2]. (**b**) Exemplar whole cell current traces in Ca^{2+} (red) and Ba^{2+} (black). CDI is seen as the faster decay of the Ca^{2+} vs. Ba^{2+} trace. Channels are now co-expressed with β_{2A} to allow examination of CDI independent of VDI effects. TS mutations confer a significant decrease in CDI (middle, right) when expressed in either relevant splice background. (**c**) Population data, the fraction of peak current remaining after 300-ms depolarization (r_{300}) is plotted for Ba^{2+} and Ca^{2+} currents. The difference between Ca^{2+} and Ba^{2+} relations at 30 mV (f_{300}) specifies the isolated effect of CDI.

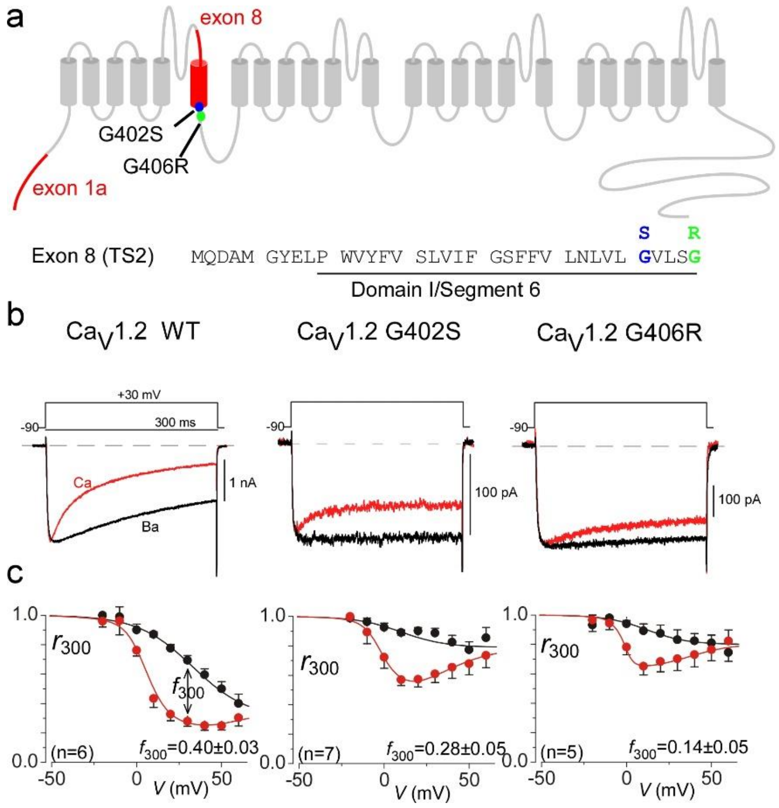

Supplementary Figure 3 | CDI effects of TS2 are conserved with an alternate beta subunit. (**a**) Diagram of TS mutations within human $Ca_V1.2$. (**b**) Exemplar whole cell current traces in Ca^{2+} (red) and Ba^{2+} (black) in the TS2 splice variant. CDI is seen as the faster decay of the Ca^{2+} vs. Ba^{2+} trace. Channels are now co-expressed with β_{1B} to allow examination of CDI in the presence of VDI. Timothy Syndrome mutations confer a significant decrease in both VDI and CDI (middle, right) (**c**) Population data, the fraction of peak current remaining after 300-ms depolarization (r_{300}) is plotted for Ba^{2+} and Ca^{2+} currents. The difference between Ca^{2+} and Ba^{2+} relations at 30 mV (f_{300}) specifies the isolated effect of CDI.

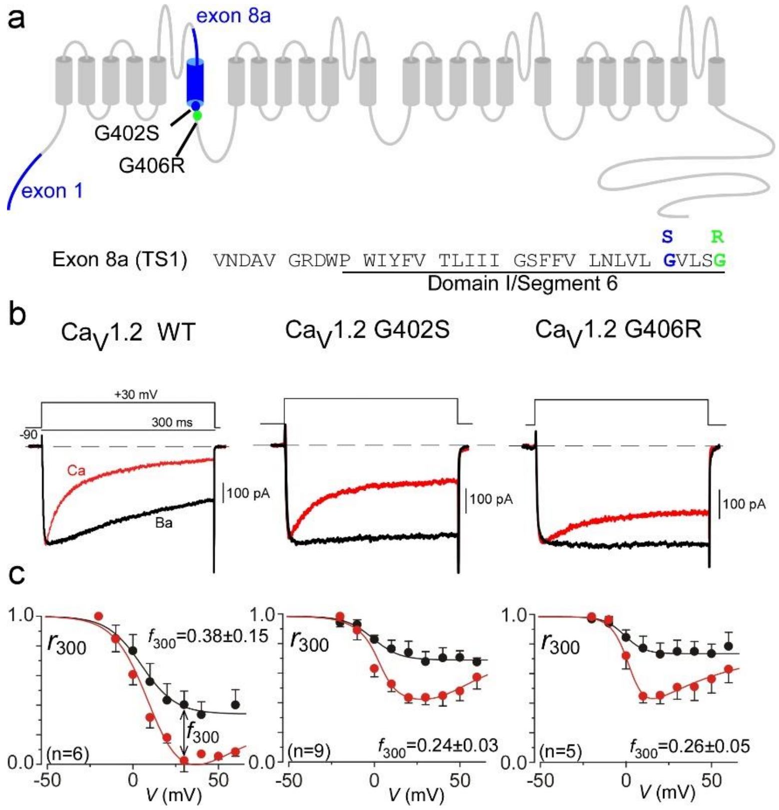

Supplementary Figure 4 | CDI effects of TS1 are conserved with an alternate beta subunit. (**a**) Diagram of Timothy Syndrome mutations within human Ca_V1.2. (**b**) Exemplar whole cell current traces in Ca^{2+} (red) and Ba^{2+} (black) in the TS1 splice variant. Channels are co-expressed with β_{1B} to allow examination of CDI in the presence of VDI. Timothy Syndrome mutations confer a significant decrease in both VDI and CDI (middle, right) (**c**) Population data, the fraction of peak current remaining after 300-ms depolarization (r_{300}) is plotted for Ba^{2+} and Ca^{2+} currents. The difference between Ca^{2+} and Ba^{2+} relations at 30 mV (f_{300}) specifies the isolated effect of CDI.

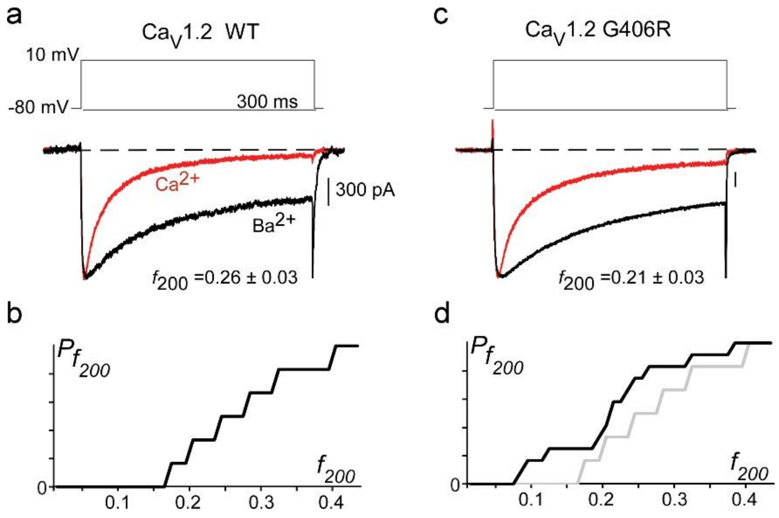

Supplementary Figure 5 | CDI effects of G406R persist in adult guinea pig ventricular myocytes (aGPVMs). (**a**) Exemplar whole cell current traces in Ca^{2+} (red) and Ba^{2+} (black) from aGPVMs. Cells are transfected with WT $Ca_V1.2$ for direct comparison with the TS transfected cells. (**b**) A cumulative histogram of the CDI metric f_{200}, showing a normal distribution centered at f_{200} = 0.26 for n = 6 cells. (**c**) Exemplar whole cell current traces for aGPVMs transfected at a low level with $Ca_V1.2$ G406R. (**d**) A cumulative histogram of f_{200} values for $Ca_V1.2$ G406R transfected myocytes from n =12 cells. Despite the low level of TS transfection, a left shift in the distribution (black) can be seen, as compared to WT channels (gray). Note that the added variability is likely due to variable (and somewhat diminutive) expression of the G406R channel. Nonetheless, an appreciable decrease in CDI can be detected.

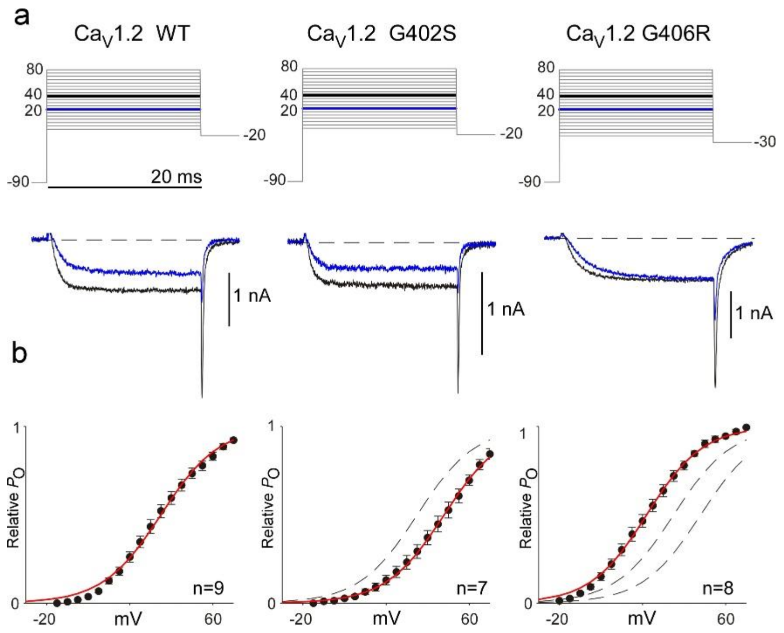

Supplementary Figure 6 | Shift in activation by tail currents. (**a**) Shifts in activation curves examined by a tail current protocol (top). Exemplar traces for 20 mV (blue) and 40 mV (black) steps. Channel is the exon1a/exon8 (TS2) backbone. (**b**) Normalized tail currents are plotted against voltage. G402S caused a significant right shift in activation (middle) compared to WT (left) with WT curve reproduced as the dashed line for comparison. G406R induced a clear left shift in activation (right) as compared to WT and G402S curves (dashed).

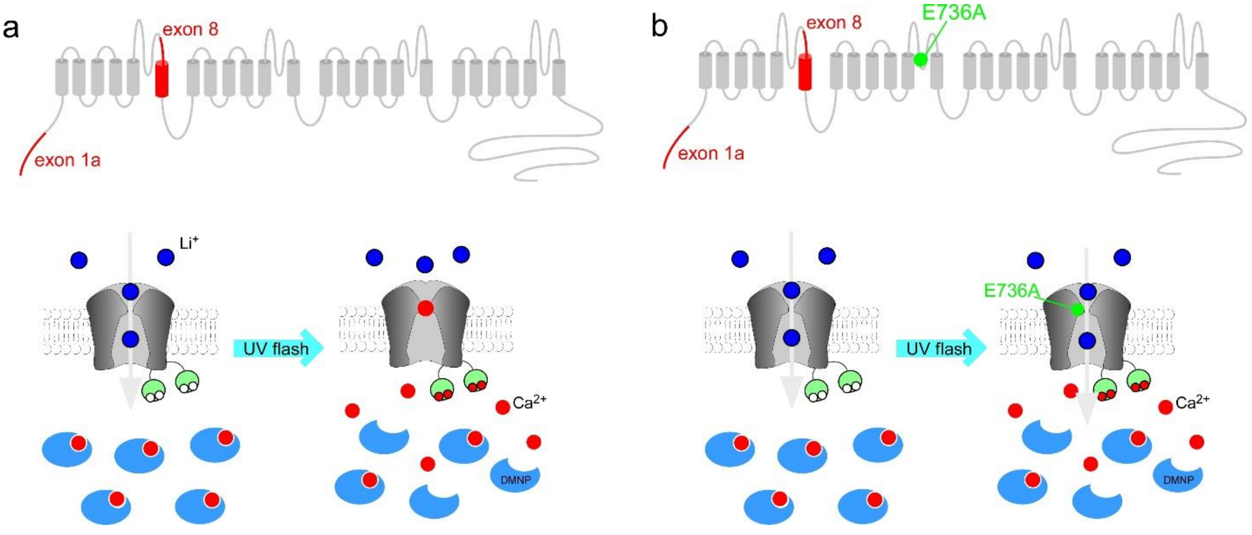

Supplementary Figure 7 | Adjusting for pore block. (**a**) Cartoon of $Ca_V1.2$ channels during a Ca^{2+} uncaging experiment. While $Ca_V1.2$ is permeant to Li^+, upon uncaging of Ca^{2+} with a UV flash, the high internal Ca^{2+} will block the entry of the less permeant Li^+ ions[3]. (**b**) The introduction of E736A mutation into $Ca_V1.2$ adjusts the permeation of $Ca_V1.2$ in favor of Li^+, thus significantly reducing the effect of Ca^{2+} pore block.

.

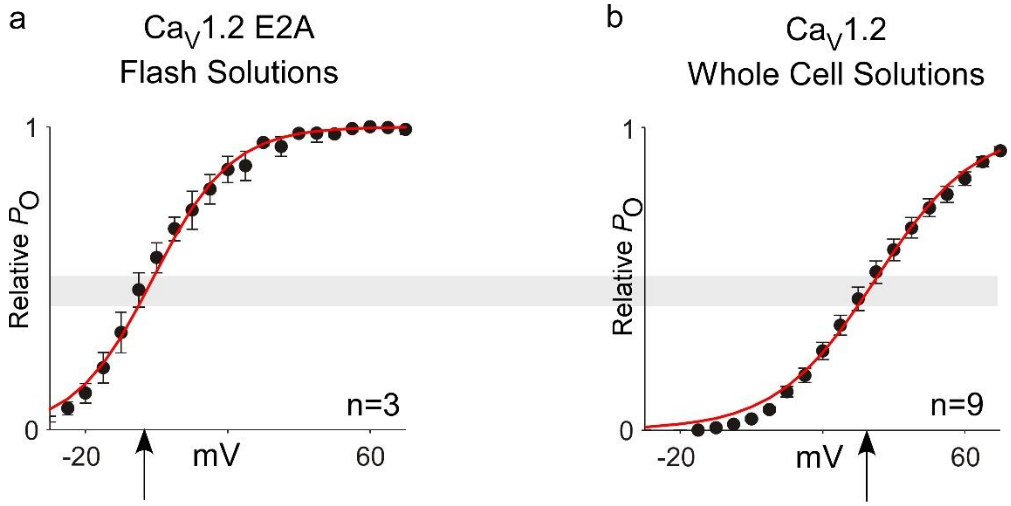

Supplementary Figure 8| Li^+ surface charge shift. (**a**) Tail current activation curves done in approximate flash solutions of 40 Li^+ and 0.5 mM EDTA. The arrow indicates the depolarizing voltage used in flash experiments. (**b**) Standard whole cell 40 Ba^{2+} external solutions and 10 mM BAPTA internal illustrating the shift in voltage dependence as compared to **a**. The arrow shows the depolarizing voltage used in whole cell experiments.

Supplementary Note 1

Adjustments to the LRd model

L-type calcium channel

The L-Type calcium channel (LTCC) module in the original LRd model was adjusted so that the duration and shape of the action potential was more similar to human. Primarily, the APD was tuned to 300 ms. Additionally, activation and inactivation equations were adjusted to match data and to allow for the introduction of TS mutant channels as a separate pool of channels. First, a CDI gate (f_{CDI}) was added for the LTCC as follows:

$$k_{on} = 4 \cdot 10^{3} \text{ mM}^{-2}\text{ms}^{-1} \tag{1}$$

$$k_{off} = 1 \cdot (K_{CDI})^{2} \cdot k_{on} \quad \text{ms}^{-1} \tag{2}$$

$$df_{CDI} = k_{on} \cdot [Ca]^{2} \cdot f_{CDI} + k_{off} \cdot (1 - f_{CDI}) \tag{3}$$

Where k_{off} is set up based on the steady state Kd of the LTCC:

$$K_{CDI} = 6.325\text{e}^{-4} \text{ mM} \tag{4}$$

The equation for df_{CDI} utilizes the assumption that the majority of CDI for the cardiac L-type channel ($Ca_V1.2$) is governed by 2 Ca^{2+} ions binding to the C-lobe of CaM, as described by the Hill coefficient of 2 determined by our Ca^{2+} uncaging experiments (Fig. 4e).

The activation gate (d), followed standard Hodgkin and Huxley equations[4], with τ_d slowed somewhat compared to the original LRd model as follows:

$$dd = \frac{d_{steadystate} - d}{\tau_d} \tag{5}$$

$$d^{1}_{steadystate} = \frac{1}{1 + e^{-(V+13)/(25/2)}} \tag{6}$$

$$d^{2}_{steadystate} = \frac{1}{1 + e^{-(V+13-15)/(25/1.5)}} \tag{7}$$

$$d_{steadystate} = d^{1}_{steadystate} \cdot d^{2}_{steadystate} \tag{8}$$

$$\tau_d = 0.59 + \frac{5}{1 + e^{-3 \cdot 0.052(V+20)+15}} \cdot \frac{1}{1 + e^{2 \cdot 0.052(V+20)-75}} \tag{9}$$

These activation parameters are matched precisely with whole cell experimental data from this study (Fig. 1). The voltage dependent inactivation gate (f) followed a similar model, but was

scaled down and slowed somewhat as compared to the originally published LRd model in order to more accurately fit experimental data:

$$df = \frac{f_{steadystate} - f}{\tau_f} \tag{10}$$

$$f_{steadystate} = 1 - 0.5 \cdot (1 - \frac{1}{1+e^{(V+32)/8}} \cdot \frac{0.8}{1+e^{(50-V)/20}}) \tag{11}$$

$$\tau_f = \frac{10}{0.0197 \cdot e^{-0.0337(V+10)^2 + .02}} \tag{12}$$

With the gates set, the total current through the L-type channel, as well as the Na^+/Ca^{2+} exchange current and Ca^{2+} activated potassium channel could now be defined as:

$$i_{Ca,Ltype} = d \cdot f \cdot (f_{CDI} + P_O^{modeCa}(1 - f_{CDI})) \cdot i_{Ca} \tag{13}$$

$$i_{Na/Ca} = d \cdot f \cdot (f_{CDI} + P_O^{modeCa}(1 - f_{CDI})) \cdot i_{Na} \tag{14}$$

$$i_{K_{Ca}} = d \cdot f \cdot (f_{CDI} + P_O^{modeCa}(1 - f_{CDI})) \cdot i_K \tag{15}$$

$$P_O^{modeCa} = 0.15 \tag{16}$$

Where $P_O{}^{modeCa}$ is a new parameter representing the relative open probability of the L-type channel in mode Ca^{2+} as compared to mode 1. i_{Ca}, i_{Na} and i_K represent the single channel open level as set by the GHK equation. This equation, as defined by the original LRd model, includes the number of channels within the permeation constants P_{Ca}, P_{Na} and P_K. For the L-type channel, this constant (P_{Ca}) was scaled by 1.1 in order to produce the desired 300 ms APD.

With the wild type channel in place, a second pool of LTCC channels were added to allow for simulation of TS mutant channels, with variable levels of expression mimicking the variable expression expected for the two variants of TS. The G406R channel was modeled with the same equations as the wild type channels, but with the following parameter changes:

$$k_{on}^{406R} = 0.1 \cdot 0.04 \cdot 10^6 \quad \text{mM}^{-2}\text{ms}^{-1} \tag{17}$$

$$K_{CDI}^{406R} = 1 \cdot 0.55 \cdot 4.62 \cdot 2.5\text{e}^{-4} \quad \text{mM} \tag{18}$$

$$d_{steadystate}^{406R1} = \frac{1}{1+e^{-(V+13)/(25/2)}} \tag{19}$$

$$d_{steadystate}^{406R2} = \frac{1}{1+e^{-(V-36-15)/(25/1.2)}} \tag{20}$$

$$\tau_d = 0.59 + \frac{19}{1+e^{-3 \cdot 0.052(V+20)+15}} \cdot \frac{1}{1+e^{2 \cdot 0.052(V+20)-75}} \tag{21}$$

$$f_{steadystate}^{406R} = 1 - 0.02 \cdot (1 - f_{steadystate}^{406R1}) \tag{22}$$

$$P_O^{406RmodeCa} = 0.6 \tag{23}$$

The G402S channel was similarly modeled such that the activation curve and VDI parameters match experimental data. The new parameters for G402S are as follows:

$$k_{on}^{402S} = 0.1 \cdot 0.04 \cdot 10^{6} \quad \text{mM}^{-2}\text{ms}^{-1} \tag{24}$$

$$K_{CDI}^{402S} = 13.8 \cdot 0.55 \cdot 4.62 \cdot 2.5\text{e}^{-4} \quad \text{mM} \tag{25}$$

$$d_{steadystate}^{402S1} = \frac{1}{1+e^{-(V+6)/(25/3)}} \tag{26}$$

$$d_{steadystate}^{402S2} = \frac{1}{1+e^{-(V-2.1+28)/(25/2.35)}} \tag{27}$$

$$\tau_{d} = 0.59 + \frac{16}{1+e^{-3\cdot 0.052(V+20)+15}} \cdot \frac{1}{1+e^{2\cdot 0.052(V+20)-75}} \tag{28}$$

$$f_{steadystate}^{402S} = 1 - 0.02 \cdot (1 - f_{steadystate}^{402S1}) \tag{29}$$

Finally, the two channel pools (WT with either G406R or G402S) were combined such that the fraction of channels expressing the TS mutation could be easily adjusted:

$$i_{Ca,Ltype}^{tot} = (1 - Fraction_{TS}) \cdot i_{Ca,Ltype} + Fraction_{TS} \cdot i_{Ca,Ltype}^{TS} \tag{30}$$

$$i_{Na/Ca}^{tot} = (1 - Fraction_{TS}) \cdot i_{Na/Ca} + Fraction_{TS} \cdot i_{Na/Ca}^{TS} \tag{31}$$

$$i_{K_{Ca}}^{tot} = (1 - Fraction_{TS}) \cdot i_{K_{Ca}} + Fraction_{TS} \cdot i_{K_{Ca}}^{TS} \tag{32}$$

Ryanodine receptors and coupling to the SR

A new module for Ca^{2+} inactivation of the ryanodine receptors (RyR) was added as follows:

$$k_{on}^{RyR} = 0.5 \cdot 10^{-3} \tag{33}$$

$$k_{off}^{RyR} = 1/10/4 \tag{34}$$

$$df_{CDI}^{RyR} = k_{on}^{RyR} \cdot {i_{Ca,Ltype}^{tot}}^{2} \cdot f_{CDI}^{RyR} + k_{off}^{RyR} \cdot (1 - f_{CDI}^{RyR}) \tag{35}$$

Here, the assumption is made that the RyR responds to the binding of 2 Ca^{2+} ions, and that the RyR inactivation is given preferential coupling to the LTCC [5, 6, 7, 8, 9], just as the activation gate (*rel*) does in the original LRd code:

$$rel = \frac{i_{Ca,Ltype} \cdot \alpha_{rel}}{(1 + \kappa_{rel}/JSR)^{9}} \tag{36}$$

$$\kappa_{rel} = 0.0625 \tag{37}$$

The NSR was also given preferential Ca^{2+} loading from the LTCC such that 90% of the Ca^{2+} entering from the LTCCs was loaded into the NSR, leaving the remaining 10% to mix into the total cytoplasmic Ca^{2+} (Ca_i):

$$\begin{aligned} dCa_i = &-(i_{Ca,nonL} + 0.1 \cdot i_{Ca,Ltype}) \cdot C_{membr} / (2F \cdot V_{myoplasm}) \\ &+ (i_{leak} - i_{SERCA}) V_{NSR} / V_{myoplasm} \\ &+ (8.28 \cdot 10^{-13} + rel \cdot (f_{CDI}^{RyR})^{4}) V_{JSR} / V_{myoplasm} \end{aligned} \tag{38}$$

$$dCa_{NSR} = i_{up} - i_{tr} \cdot V_{JSR} / V_{NSR} - i_{leak} - 0.9 \cdot i_{Ca,Ltype} \cdot C_{membr} / 2FV_{NSR} \quad (39)$$

$$dCa_{JSR} = i_{tr} - rel \cdot (f_{CDI}{}^{RyR})^4 \quad (40)$$

Note that the RyR now has 4 inactivation (*f*) gates.

NSR Ca^{2+} handling was also adjusted so as to accommodate the increased Ca^{2+} entry due to the longer APD (300ms), as well as the preferential loading of the NSR by the LTCC:

$$i_{sarcolema} = 5.1750 \cdot Ca_i / (k_{SERCA} + Ca_i) \quad (41)$$

$$i_{Ca,bkgr} = g_{Ca,bkgr} \cdot (V - \log(Ca_0 / Ca_i) / 2(F / RT) \quad (42)$$

$$i_{leak} = .00875 / Ca_{NSR} \cdot 5 \cdot NSR / (NSR + 5) \quad (43)$$

$$Ca_{NSR} = 15 \quad (44)$$

$$i_{SERCA} = .00875 \cdot Ca_i / (Ca_i + k_{SERCA}) \quad (45)$$

The current flux from the NSR to the JSR (i_{tr}) was also adjusted such that restitution did not occur until after repolarization of the action potential[10].

$$i_{tr} = i(NSR - JSR) / \tau_{tr} \qquad V < -30 \quad (46)$$

$$i_{tr} = 0 \qquad V > -30 \quad (47)$$

Lastly, the instantaneous Ca^{2+} buffering was somewhat adjusted to prevent non-physiological overload of Ca^{2+} during the lengthened action potential. The buffering capacities were increased by a factor of 2.5 resulting in:

$$CMD_{buffer} = 1.25 \text{ mM} \quad (48)$$

$$TRPN_{buffer} = 0.175 \text{ mM} \quad (49)$$

All other equations in the model not mentioned here were left as in the original published 2007 version[1111]

Supplementary References

1. Liao P, Soong TW. CaV1.2 channelopathies: from arrhythmias to autism, bipolar disorder, and immunodeficiency. *Pflugers Arch* **460**, 353-359 (2010).

2. Liao P, Yong TF, Liang MC, Yue DT, Soong TW. Splicing for alternative structures of Cav1.2 Ca2+ channels in cardiac and smooth muscles. *Cardiovasc Res* **68**, 197-203 (2005).

3. Ellinor PT, Yang J, Sather WA, Zhang JF, Tsien RW. Ca2+ channel selectivity at a single locus for high-affinity Ca2+ interactions. *Neuron* **15**, 1121-1132 (1995).

4. Hodgkin AL, Huxley AF. A quantitative description of membrane current and its application to conduction and excitation in nerve. *The Journal of physiology* **117**, 500-544 (1952).

5. Antoni H. Elementary events in excitation-contraction coupling of the mammalian myocardium. *Basic Res Cardiol* **72**, 140-146 (1977).

6. Antoni H, Jacob R, Kaufmann R. [Mechanical response of the frog and mammalian myocardium to changes in the action potential duration by constant current pulses]. *Pflugers Arch* **306**, 33-57 (1969).

7. Fabiato A. Calcium-induced release of calcium from the cardiac sarcoplasmic reticulum. *Am J Physiol* **245**, C1-14 (1983).

8. Fozzard HA. Heart: excitation-contraction coupling. *Annu Rev Physiol* **39**, 201-220 (1977).

9. Marban E, Wier WG. Ryanodine as a tool to determine the contributions of calcium entry and calcium release to the calcium transient and contraction of cardiac Purkinje fibers. *Circulation research* **56**, 133-138 (1985).

10. Burkhoff D, Yue DT, Franz MR, Hunter WC, Sagawa K. Mechanical restitution of isolated perfused canine left ventricles. *Am J Physiol* **246**, H8-16 (1984).

11. Livshitz LM, Rudy Y. Regulation of Ca2+ and electrical alternans in cardiac myocytes: role of CAMKII and repolarizing currents. *American journal of physiology* **292**, H2854-2866 (2007).
